# Data on effect of variety, seedling transplanting age and nitrogen fertilizer rates on growth performance of rice in Southern Guinea Savannah, Nigeria

**DOI:** 10.1016/j.dib.2021.107582

**Published:** 2021-11-17

**Authors:** Simon Nda, Erick Sebetha

**Affiliations:** Food Security and Safety Niche Area, Faculty of Natural and Agricultural Sciences, North West University, Mafikeng Campus, Private Bag X 2046, Mmabatho, 2738 Nigeria

**Keywords:** Location, Nitrogen fertilizer rates, Rice and seedling transplanting age

## Abstract

The age of rice seedling at transplanting and application of nitrogen fertilizer are important husbandry practices for improved rice growth. The data showed the effect of variety, seedling transplanting age and nitrogen fertilizer rates on the growth of rice. Agronomic field trial was conducted during 2015 and 2016 planting seasons at National Cereals Research Institute Farms of Edozhigi and Badeggi located in the Southern Guinea Savannah of Nigeria. To assessed the effect of variety, seedling transplanting age and nitrogen fertilizer rates on the growth of rice. Treatments were laid out as split–split plot arrangement fitted into Randomized Complete Block Design (RCBD) with three replications. The main plot consisted of two rice varieties (FARO 44 and FARO 52), four seedling transplanting ages (7, 14, 21 and 28 days after planting) and five inorganic N fertilizer rates (0, 60, 120, 180 and 240 kg N/ha) constituted the sub-plot and sub-sub plot respectively. Growth data viz plant height, number of tillers and leaf area index were measured at 30 and 45 days after transplanting (DAT) during the two planting seasons. Plant height was taken from five randomly selected and tagged plants from ground surface to the tip of the leaf or panicle of the main stem using measuring tape. The number of tillers per plant was counted from five randomly selected and tagged hills in each plot from two middle rows. .The leaf area (cm^2^) of the leaves was obtained from five randomly selected tagged plants by measuring the leaf length from the base to the tip with a ruler also the widest part of the leaf. The leaf area (LA) was estimated using the equation:

LA = length × breath × 0.75 [1].

The leaf area value obtained was used to calculate the leaf area index (LAI), which describes the efficiency of the photosynthetic process on the photosynthetic surface and calculated as the total leaf area/ground area. Data collected were subjected to one way analysis of variance (ANOVA) using GenSTAT 11^th^ edition and the difference between treatments mean tested using least significant difference (LSD) at 5% probability level. The data are of value to agronomists in search of reference. It has the potential to be used to predict seedling age of rice at transplanting and the appropriate nitrogen fertilizer rate for rice growth.

## Specifications Table

**Table:** 

Subject	Agronomy
Specific subject area	The data provides baseline information on crop production, crop nutrition and soil fertility
Type of data	Table Figure
How data were acquired	The data were acquired through field work. Plant height was measured from the base of the plant to the tip of the tagged stands using meter rule The number of tillers was counted from tagged stands and recorded The leaf area (LA) was calculated using the equation: LA= length × breath × 0.75 [1] from the tagged stands
Data format	Raw Analyzed
Parameters for data collection	Plant height at 30 and 45 days after transplanting, number of tillers per stand at 30 and 45 days after transplanting, and leaf area index at 30 and 45 days after transplanting
Description of data collection	Growth data were measured at 30 and 45 days after transplanting (DAT). Plant height was taken from the base of the plants from ground surface to the tip of the leaf or panicle of the main stem using measuring tape. The number of tillers per plant was counted from five randomly selected and tagged hills in each plot from two middle rows. The leaf area (cm^2^) of the leaves was obtained from five randomly selected tagged plants by measuring the leaf length from the base to the tip with a ruler also the widest part of the leaf. The leaf area (LA) was estimated using the equation: LA = length × breath × 0.75 [1]
Data source location	Institution: National Cereal Research Institute Badeggi, Nigeria City/Town/Region: Badeggi Country: Nigeri Latitude and longitude: Edozhigi (9^⁰^ 05^1^ N; 5^⁰^ 57^1^ E) [2] and Badeggi (9^⁰^ 45^1^ N; 6^⁰^ 07^1^ E) [3]
Data accessibility	Nda, Simon (2021), “Article”, Mendeley Data, V2, https://doi.org/10.17632/bpnpc5vyjv.2 https://data.mendeley.com/datasets/bpnpc5vyjv/

## Value of the Data

• Soil nutrient depletion and inappropriate nitrogen fertilizer use has been responsible for the low growth of rice in Nigeria. Hence, the present data are important as it revealed the effect of variety, seedling transplanting age and nitrogen fertilizer rates on rice growth.
• These data will be of tremendous benefit to agronomist, crop nutritionist and soil scientists.
• The data could be used as a reference factor to broaden comparable experiment with a purpose to give similar insight to the effect of variety, nitrogen fertilizer rates and the appropriate age of transplanting rice seedlings.

## 1 Data Description

### 1.1 Description of raw data

The supplementary data indicates the raw dataset of plant height, number of tillers and leaf area index at 30 and 45 days after transplanting for 2015 and 2016 planting seasons. (Exel sheet 1).

### 1.2 Plant height

Plant height was taken from the base of the plants from ground surface to the tip of the leaf or panicle of the main stem using measuring tape at 30 and 45 days after transplanting in 2015 and 2016 planting seasons for the two varieties viz- FARO 52 (variety I) and FARO 44 (variety II). Raw data was collected for the three replications at the two locations viz- Badeggi (site i) and Edozhigi (site ii).

### 1.3 Number of tillers

The number of tillers per plant was counted from five randomly selected and tagged hills in each plot from two middle rows. The raw data was recorded for the three replications and the two locations viz Badeggi (site i) and Edozhigi (siteii) during 2015 and 2016 planting seasons.

### 1.4 Leaf area index

The leaf area (cm^2^) of the leaves was obtained from five randomly selected tagged plants by measuring the leaf length from the base to the tip with a ruler also the widest part of the leaf. The leaf area value obtained was used to calculate the leaf area index (LAI), which describes the efficiency of the photosynthetic process on the photosynthetic surface and calculated as the total leaf area/ground area. Raw data was collected for the two planting seasons 2015 and 2016. The raw data was recorded for the three replications and the two locations.

The effect of treatment factors (location, nitrogen fertilizer rates, seedling transplanting age and variety) on plant height at 30 and 45 days after transplanting in Badeggi and Edozhigi during 2015 and 2016 planting seasons were presented in Table 1.

**Table 1:** The effect of location, nitrogen fertilizer rates, seedling transplanting age and variety on rice plant height (cm) during 2015 and 2016 planting seasons.

	2015		2016	
Treatment factors	Plant height (cm) 30 DAT	Plant height (cm) 45 DAT	Plant height (cm) 30 DAT	Plant height (cm) 45 DAT
**Location**				
Badeggi	41.348⁎⁎⁎	48.39⁎⁎⁎	28.55	70.78⁎⁎⁎
Edozhigi	36.98	43.26	34.44⁎⁎⁎	41.65
SEM	0.544	0.738	0.330	0.413
LSD _(0.05)_	1.52	2.06	0.92	1.15
**Nitrogen rate**				
0 kg/ha	38.00	44.06	30.90	55.22
60 kg/ha	38.07	44.45	31.03	55.51
120 kg/ha	40.89	48.14	31.04	56.37
180 kg/ha	39.23	45.76	31.54	55.86
240 kg/ha	39.62	46.70	32.96*	58.11*
SEM	0.860	1.167	0.522	0.653
LSD _(0.05)_	2.4	3.26	1.46	1.83
**Transplanting age**				
7 days	34.91	42.02	26.65	49.52
14 days	36.87	42.76	29.86	54.55
21 days	41.35	47.15	33.67	58.84
28 days	43.51⁎⁎⁎	51.36⁎⁎⁎	35.79⁎⁎⁎	61.95⁎⁎⁎
SEM	0.769	1.044	0.467	0.584
LSD _(0.05)_	2.15	2.92	1.30	1.63
**Variety**				
Faro 52	38.19	47.53*	34.70*	64.61*
Faro 44	40.14*	44.11	28.29	47.82
SEM	0.544	0.738	0.330	0.413
LSD _(0.05)_	1.52	2.06	0.92	1.15

DAT= days after transplantin; SEM = standard error of means.

LSD = Least significant difference.

⁎ = significant at *p* < 0.05.

⁎⁎⁎ = significant at *p* < 0.001.

Table 2 exhibits the influence of each treatment factors on number of tillers at 30 and 45 days after transplanting in Badeggi (site i) and Edozhigi(site ii) during 2015 and 2016 planting seasons.

**Table 2:** The effect of location, nitrogen fertilizer rate, seedling transplanting age and variety on rice number of tillers during 2015 and 2016 planting seasons.

	2015		2016	
Treatment factors	Number of tillers 30 DAT	Number of tillers 45 DAT	Number of tillers 30 DAT	Number of tillers 45 DAT
**Location**				
Badeggi	12.12	14.95	5.62	13.68
Edozhigi	14.97⁎⁎⁎	17.64⁎⁎⁎	17.15⁎⁎⁎	17.88⁎⁎⁎
SEM	0.276	0.384	0.371	0.265
LSD _(0.05)_	0.77	1.07	1.04	0.74
**Nitrogen rates**				
0 kg	13.62	14.08	9.83	14.17
60 kg	13.63	15.51	11.31	15.42
120 kg	14.55*	17.95*	12.16	15.84
180 kg	13.57	17.48	12.25*	16.69*
240 kg	12.35	16.46	11.37	16.23
SEM	0.437	0.607	0.587	0.419
LSD _(0.05)_	1.22	1.70	1.64	1.17
**Transplanting age**				
7 days	12.13	15.86	12.00	12.70
14 days	13.35	15.94	10.02	14.68
21 days	14.05	17.02	10.96	16.43
28 days	14.65⁎⁎⁎	16.37	12.55⁎⁎⁎	19.29⁎⁎⁎
SEM	0.390	0.543	0.525	0.375
LSD _(0.05)_	1.10	1.52	1.47	1.05
**Variety**				
Faro 52	14.96*	17.80*	10.75	13.23
Faro 44	12.13	14.79	12.02*	18.32*
SEM	0.276	0.384	0.371	0.265
LSD _(0.05)_	0.77	1.07	1.04	0.74

DAT= days after transplanting.

SEM=standard error means.

LSD = Least significant difference.

⁎ = significant at *p* < 0.05.

⁎⁎⁎ = significant at *p* < 0.001.

The effect of treatment factors on leaf area index at 30 and 45 days after transplanting at Badeggi (site i) and Edozhigi (site ii) during 2015 and 2016 planting seasons were presented in Table 3.

**Fig. 1a:**
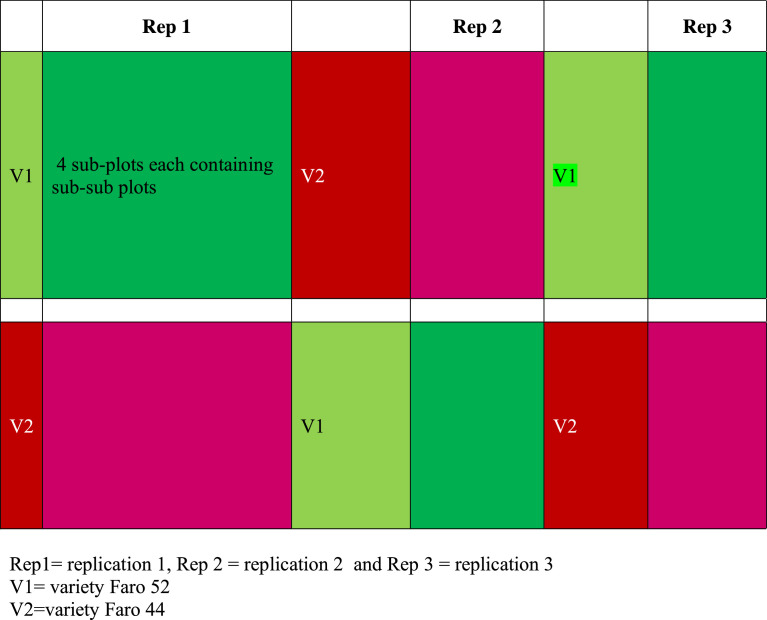
The main field layout of variety.

**Fig. 1b:**
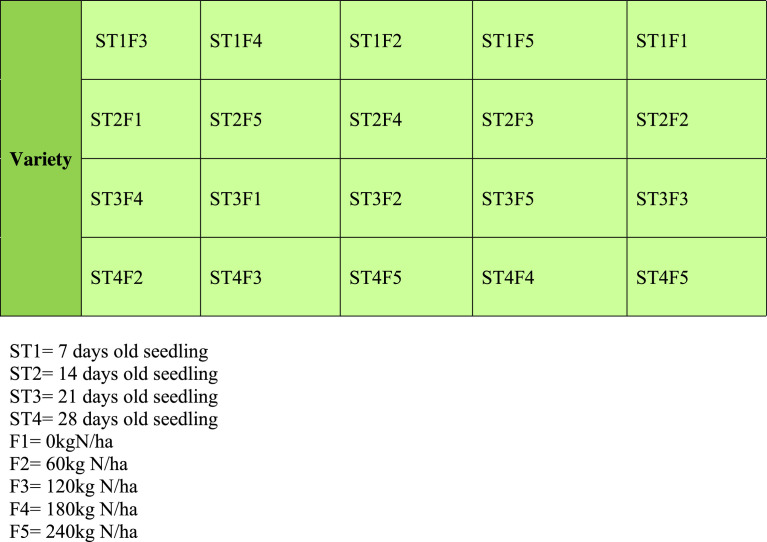
The sub- plot field layout of seedling transplanting age and nitrogen fertilizer rates.

**Table 3:** The effect of location, nitrogen fertilizer rates, seedling transplanting age and variety on rice leaf area index during 2015 and 2016 planting seasons.

	2015		2016	
Treatment factors	Leaf area index 30 DAT	Leaf area index 45 DAT	Leaf area index 30 DAT	Leaf area index 45 DAT
**Location**				
Badeggi	3.32	4.09	2.19	4.78
Edozhigi	3.44	3.98	9.50⁎⁎⁎	11.31⁎⁎⁎
SEM	0.0908	0.0972	0.501	0.265
LSD _(0.05)_	0.25	0.27	1.40	0.74
**Nitrogen rates**				
0 kg	3.34	3.96	5.55	8.66
60 kg	3.25	4.03	5.61	6.45
120 kg	3.52	4.12	6.57	6.22
180 kg	3.43	3.99	5.83	9.29
240 kg	3.36	4.07	5.68	9.60⁎⁎⁎
SEM	0.1436	0.1536	0.792	0.419
LSD _(0.05)_	0.40	0.43	2.21	1.17
**Transplanting age**				
7 days	2.69	3.50	3.72	4.79
14 days	3.08	3.61	4.94	6.14
21 days	3.69	4.35	7.48	11.34⁎⁎⁎
28 days	4.06⁎⁎⁎	4.68⁎⁎⁎	7.52⁎⁎⁎	9.90
SEM	0.1284	0.1374	0.708	0.375
LSD _(0.05)_	0.36	0.38	1.99	1.05
**Variety**				
Faro 52	3.80⁎⁎⁎	4.69⁎⁎⁎	4.70	8.94⁎⁎⁎
Faro 44	2.96	3.38	7.00⁎⁎⁎	7.15
	0.0908	0.0972	0.501	0.265
LSD _(0.05)_	0.25	0.27	1.40	0.74

DAT= days after transplanting.

SEM=standard error means.

* = significant at *p* < 0.05.

LSD = Least significant difference.

⁎⁎⁎ = significant at *p* < 0.001.

## 2 Experimental Design, Materials and Methods

A 2-year agronomic field experiment was planted at two locations Edozhigi (9^⁰^ 05^1^ N; 5^⁰^ 57^1^ E) [1]. and Badeggi (9^⁰^ 45^1^ N; 6^⁰^ 07^1^ E) [3] under lowland ecology at the National Cereals Research Institute (NCRI) Farms between the month of June to October during 2015 and 2016 planting seasons. The soil at Edozhigi is silt-loam, iron-toxic and classified as dystricgleysol [4]. On the other hand,soils at Badeggi are sandy loam with bulk density of 1.489 g m^.3^ and classified as ultisol (Ayotade and Fagade, 1993). Both sites are located in the Southern Guinea Savannah zone of Nigeria where climate is characterized by a distinct wet season from April to November and dry season from November to March. The relative humidity at dry and raining season ranges between 75–85%. Annual rainfall at the sites ranges between 1,200–1,400 mm and mean temperature of between 23^–^33 ^⁰^C.

The experimental design indicated that the experiment had three replications in each location. At each site, it was laid out in a split–split plot arrangement fitted into Randomized Complete Block Design (RCBD) as illustrated in Figs. 1a and 1b.

Treatments consisted of two rice varieties (FARO 52 and FARO 44), four seedling transplanting ages (7, 14, 21 and 28 days after sowing, DAS) and five inorganic N fertilizer rates (0, 60, 120, 180 and 240 kg N/ha). Rice varieties were allocated to the main plot while seedling transplanting age and nitrogen fertilizer rates constituted the sub plot and sub-sub plot, respectively. The size of each main plot was 193.8 m^2^ while the sub plot and sub-sub plot were 34.2 m^2^ and 5.4 m^2^, respectively. Each sub-sub plot had a dimension of 3 x 1.8 m with alley way of 2.0 m between the main plots, 1.0 m between sub plot, and 1 m between replications. Hence, there were a total of 20 sub-sub plots per replication which gave a total of 60 plots per variety and a total of 120 plots with experimental plot size of 2,184 m^2^ for each site.

Basal application of inorganic P and K fertilizers using single super phosphate (SSP) and murate of potash (MOP), respectively were both applied at 40 kg/ha. Nitrogen fertilizer (urea 46% N) was split applied to the experimental plots based on each treatment with half of the various inorganic N fertilizer (urea) rates applied together with the P (single super phosphate SSP) and K (murate of potash) prior to transplanting and manually worked into the soil using hoe while the remaining half was broadcasted at panicle initiation. The flooded field was drained before transplanting. Two rice seedlings were transplanted per hill at a spacing of 20 × 20 cm between and within rows respectively, resulting in a total of 16 rows per plot. The field was flooded after transplanting. Regular weeding was undertaken in the experimental plot using hoe. The experiment was executed for two planting seasons. The data obtained was analysed using GenStat 11^th^ edition (2008). Data were subjected to one way analysis of varience and LSD was used to compare the mean of the data for significant differences at *p* < 0.05.

## Ethics Statement

Oryza sativa plants were used in this study. Two varieties of Faro 44 and Faro 52 were involved in the experiment

## CRediT Author Statement

**Simon Nda:** Conceptualization, Methodology, Writing – original draft; **Erick Sebetha:** Software, Writing – review & editing.

## Declaration of Competing Interest

The authors declare that they have no known competing financial interests or personal relationships which have or could be perceived to have influenced the work reported in this article.
